# Systemic Inflammation as a Modulator of FcRn-dependent IgG Pharmacokinetics: Implications for Broadly Neutralising Antibody Efficacy in HIV Prevention

**DOI:** 10.1007/s11904-026-00791-2

**Published:** 2026-07-10

**Authors:** Andile Khumalo, Sanjali Pillay, Jivanka Mohan, Sharana Mahomed, Derseree Archary

**Affiliations:** 1 Centre for the AIDS Programme of Research in South Africa (CAPRISA), University of KwaZulu-Natal, Durban, 4001 South Africa; 2 Department of Medical Microbiology, University of KwaZulu-Natal, Durban, 4001 South Africa

**Keywords:** Broadly neutralising antibodies, HIV prevention, Neonatal fragment crystallisable receptor, Pharmacokinetics, Sub-Saharan Africa, Systemic inflammation

## Abstract

**Purpose of review:**

Broadly neutralising antibodies (bNAbs) represent a promising long-acting modality for HIV prevention. However, substantial interindividual pharmacokinetic variability poses a threat to real-world efficacy. This review investigates the impact of systemic inflammation, driven by the high prevalence of sexually transmitted infections (STIs), co-endemic pathogens, and metabolic activation, on bNAb persistence, particularly within the vulnerable demographic of women in sub-Saharan Africa. We aim to bridge the gap between neonatal fragment crystallisable receptor (FcRn) biology and clinical pharmacokinetic observations to identify why certain individuals clear bNAbs faster than others.

**Recent findings:**

Synthesis of recent clinical trial data and cross-therapeutic PK evidence indicates that systemic inflammatory biomarkers are strong predictors of accelerated monoclonal antibody clearance. Recent evidence suggests that chronic inflammation modulates bNAb half-life through the transcriptional downregulation of the *FCGRT* gene, which encodes FcRn, and the competitive saturation of FcRn recycling pathways. These findings suggest that bNAbs’ standard dosing does not account for the inflammatory burden that characterises many real-world populations, potentially leading to sub-therapeutic concentrations and an increased risk of breakthrough HIV infection.

**Summary:**

The host’s inflammation status is a primary and currently overlooked determinant of antibody pharmacokinetics. This review provides a roadmap to overcoming the pharmacokinetic heterogeneity modulated by systemic inflammation through establishing the link between inflammation and increased antibody clearance. Bridging these insights paves the way for effective bNAbs with promising implications for prospective HIV prevention and cure strategies that are not only effective in controlled clinical trials but also protective in real-world settings.

**Supplementary Information:**

The online version contains supplementary material available at 10.1007/s11904-026-00791-2.

## Introduction

Broadly neutralising antibodies (bNAbs) represent a promising long-acting HIV prevention modality that complements pre-exposure prophylactic strategies. Their prophylactic efficacy depends on sustaining systemic concentrations above the predicted 80% serum neutralisation titre (PT80), governed by the neonatal Fc receptor (FcRn), which rescues IgG from lysosomal degradation [1, 2]. Leading bNAb candidates like 3BNC117 (V3 glycan), CAP256V2LS (V1V2 glycan), VRC07-523LS (CD4 binding site), and 10-1074LS (CD4 binding site) have demonstrated favourable safety profiles and antiviral activity in early-phase clinical trials [3–5]. However, substantial interindividual variability in bNAb pharmacokinetics has been observed, and it remains a significant barrier to achieving consistent prophylactic efficacy.

Clinical trial data from ongoing HIV prevention clinical trials are beginning to substantiate the hypothesis that systemic inflammation modulates monoclonal antibodies (mab) pharmacokinetics. A post-hoc analysis of the antibody-mediated prevention (AMP) trial provided key evidence for this, demonstrating accelerated VRC01 clearance in participants receiving the bNAb while on oral pre-exposure prophylaxis (PrEP), which correlated with elevated intestinal fatty acid binding protein (I-FABP), a marker of gut barrier injury and an activated immune state [6]. Concurrently, a sub-study of the CAPRISA 012B trial identified transient lymphocytopenia and elevated levels of TNF-α, IL-6, and IFN-γ following administration of VRC07-523LS and CAP256V2LS in participants with lower baseline immunoglobulin subclass 1 (IgG1) levels [7]. These findings suggest that substantial inter-individual variability, based on pre-existing immune profiles, may predispose certain individuals to increased mAbs clearance. This complexity highlights that the relationship between inflammation and FcRn regulation is multifaceted and context-dependent.

Recent data from murine models and inflammatory disease studies suggest that the host’s systemic inflammatory state may significantly alter bNAb pharmacokinetics by disrupting cellular FcRn expression and IgG recycling efficiency [8–10]. In sub-Saharan Africa (SSA), the populations who stand to benefit from long-acting bNAb prevention include adolescent girls and young women (AGYW), pregnant women at risk of mother-to-child transmission, and HIV-exposed uninfected (HEU) infants during the breastfeeding window. These groups are also those in whom co-endemic infections, sexually transmitted infections (STIs), and metabolic immune activation converge [11–13]. These factors sustain elevated interleukin-1 beta (IL-1β), IL-6, interferon-gamma (IFN-γ), and tumour necrosis factor alpha (TNF-α). These inflammatory cytokines dysregulate FcRn expression and traffic antibodies from recycling to degradation pathways [9, 10, 14]. However, inflammatory status remains systematically unaccounted for in current bNAb dosing strategies.

This review synthesises clinical trial evidence, FcRn biology, and cross-therapeutic pharmacokinetic data to establish that systemic inflammatory biomarkers predict therapeutic IgG clearance across disease contexts and propose their integration into bNAb trial designs. This review characterises the unique inflammatory burden in SSA populations and identifies accessible biomarkers, including high-sensitivity C-reactive protein (hs-CRP), serum albumin and Fc fragment of the IgG receptor and transporter (FCGRT) genotyping that could predict bNAb clearance. By bridging these insights, we propose a framework for prospective validation studies and the integration of biomarker-guided dosing algorithms into future clinical trial designs in high-burden settings. Findings from this review have translational implications for HIV prevention studies actively enrolling participants across SSA, where diminished bNAb efficacy could result in breakthrough HIV infections and undermine global prevention efforts.

## Clinical Evidence for Inflammation-mediated Pharmacokinetic Variability Across Therapeutic Fields

The hypothesis that systemic inflammation is an essential yet an under-characterised determinant of mAbs is not novel to the field of HIV prevention. Emerging clinical trial data provide direct evidence that inflammatory biomarkers correlate with accelerated antibody clearance and reduced efficacy. This section synthesises evidence from HIV prevention bNAb clinical trials (Supplementary Table 1) and studies across other mAbs therapeutics (Table 1) to demonstrate the clinical relevance of pharmacokinetic variability and its association with systemic inflammation.

**Table 1:** Cross-therapeutic evidence for inflammation-mediated monoclonal antibody pharmacokinetic variability

Therapeutic field(s)	Monoclonal antibodies	Inflammatory biomarker correlate	Magnitude of clearance Increase	Clinical consequence	Lesson for bNAb prevention field
**Oncology** (Non-small cell lung cancer, mesothelioma)	Nivolumab, pembrolizumab	CRP, ECOG performance status, LDH, serum albumin	Albumin < 37.5 g/L linked to > 20% increase in nivolumab CL [15]. Albumin < 35 g/L linked to ~ 25% increase in pembrolizumab CL [16, 17].	Reduced AUC predicts primary non-response and overall survival in non-small cell lung cancer and mesothelioma. Residual PK variability not explained by tumour genetics.	Exposure-targeted dosing is approved for pembrolizumab; HIV trials lack similar biomarker-stratified strategies despite shared FcRn mechanisms
**Oncology** (B-cell malignancies)	Obinutuzumab	CD19/CD20 target antigen burden, CRP; serum albumin	TMDD is the primary driver; CRP/Albumin are independent secondary covariates [18]	Incomplete B-cell depletion and reduced remission duration in high-disease-activity patients. Standard weight-based dosing does not correct suboptimal exposure.	In HIV-negative prevention, TMDD is absent, there is no viral antigen load to form clearance-accelerating immune complexes. FcRn-mediated mechanisms therefore account bNAb PK variability
**Autoimmune conditions** (Inflammatory bowel disease, rheumatoid arthritis)	Anti-TNF-α: infliximab, adalimumab; Anti-IL-6R: tocilizumab	CRP, Albumin, Faecal Calprotectin, FCGRT VNTR	2–4 fold higher CL in active vs. remission IBD. Mucosal inflammation leads to increased faecal loss of infliximab [19–21].	Subtherapeutic concentrations lead to PK failure in 30–40% of patients within 12 months.	TDM is the standard of care. High-clearance phenotypes in IBD are quantitatively similar to the chronic inflammation seen in SSA AGYW.
**Autoimmune conditions** (Myasthenia gravis)	Efgartigimod	Total serum IgG (FcRn saturation marker), FCGRT VNTR genotype, serum albumin	Efgartigimod drives 50–75% reduction in total IgG within 4–8 weeks [22]. VNTR2/3 correlates with higher IVIg non-response [23].	Approved clinical use of FcRn inhibitors to clear pathogenic IgG constitutes a proof-of-concept for the entire FcRn-bNAb-inflammation axis.	High endogenous IgG in SSA mimics partial FcRn blockade, competing with bNAbs for recycling. This has similar implications for HIV bNbs .
**Infectious diseases** (COVID-19)	Casirivimab/imdevimab, bamlanivimab/etesevimab, tixagevimab/cilgavimab	IL-6, CRP, serum albumin, D-dimer	Significantly reduced exposure in severe vs. mild COVID. D-dimer/albumin emerged as independent PK covariates [24].	Reduced viral clearance and attenuated clinical benefit in high-inflammatory states.	Chronic LGSI in SSA may produce a persistent “PK erosion” more impactful than transient acute illness
**Infectious diseases** (Respiratory syncytial virus)	Nirsevimab	Body weight ADA, gestational age, disease severity	Nirsevimab (YTE-modified) showed weight-dependent CL. Fixed 50 mg dose was suboptimal in infants > 5 kg (Phase 2b), necessitating weight-banded dosing [25].	Exposure response modelling drives approved weight-banded dosing for nirsevimab.	Demonstrates that even for prophylactic mAbs in low-inflammation settings, biological covariates (body weight, disease severity) modulate PK failure at fixed doses, necessitating exposure-guided dosing.
**FCGRT Pharmacogenomic**s	Infliximab	37-bp VNTR (VNTR2 vs. VNTR3); hsa-miR-3181	VNTR3/3 has higher FcRn expression than VNTR2/3 [26]. Infliximab troughs: 7.00 vs. 4.14 µg/mL (3/3 vs. 2/3) [27].	VNTR2/3 carriers have higher IVIg non-response and require more frequent SC dosing (SCIg).	FCGRT VNTR frequencies in SSA are uncharacterised. Major pharmacogenomic gap for HIV bNAb deployment.

Abbreviations: *ADA* anti-drug antibodies, *AGYW* adolescent girls and young women, *AUC* area under the curve, *bNAb*  broadly neutralising antibody, *CL* clearance, *CRP* C-reactive protein, *ECOG* Eastern Cooperative Oncology Group, *FcRn* neonatal Fc receptor, *HIV* human immunodeficiency virus, *IBD* Inflammatory bowel disease, *IgG* Immunoglobulin G, *IL-6* Interleukin-6, *IL-6R* Interleukin-6 receptor, *IVIg* Intravenous immunoglobulin, *LDH* lactate dehydrogenase, *LGSI* low-grade systemic inflammation, *mAb* monoclonal antibody, *miR* microRNA, *PK* pharmacokinetics, *SC* subcutaneous, *SCIg* subcutaneous immunoglobulin, *SSA* sub-Saharan Africa, *TDM* therapeutic drug monitoring, *TMDD* target-mediated drug disposition, *TNF-α* tumor necrosis factor alpha, *VNTR* variable number tandem repeat

### Evidence from HIV Prevention Clinical Trials

#### The Antibody-mediated Prevention Clinical Trial: Mucosal Barrier Disruption Accelerates VRC01 Clearance

The AMP HVTN 704/HPTN 085 and HVTN 703/HPTN 081 clinical trials represented the first large-scale evaluation of bNAb-based HIV prevention [28]. These clinical trials enrolled over 4,600 participants across the Americas, Europe, and SSA, who received intravenous infusions of VRC01 every eight weeks. While the trials established “proof-of-concept” for bNAb prevention, they also revealed substantial pharmacokinetic heterogeneity that could not be explained by body weight or dosing regimen alone. A post-hoc analysis of the AMP clinical trial identified a positive correlation between serum I-FABP, a marker of intestinal epithelial permeability, and accelerated VRC01 clearance in PrEP (tenofovir-emtricitabine) users [6].

In PrEP users, VRC01 clearance was 0.08 L per day faster, which corresponded to dose-normalised area under the concentration-time curve (AUC) that was 0.29 day/mL lower [6]. Pharmacokinetic modelling predicted a 14% reduced prevention efficacy against VRC01-sensitive strains, a clinically significant reduction that, in a real-world prevention context, increases the risk of breakthrough HIV infection [6]. This finding is consistent with inflammation-mediated FcRn dysregulation. I-FABP elevation demonstrates intestinal epithelial injury, which triggers local and systemic inflammatory responses characterised by elevated IL-6, TNF-α, and IFN-γ [29].

These cytokines suppress FCGRT transcription in intestinal epithelial cells and systemically in vascular endothelium, thereby reducing FcRn-mediated rescuing of VRC01 from lysosomal degradation [14, 30]. The association with I-FABP was independent of HIV viral load and CD4 T cell count, indicating that mucosal immune activation rather than HIV infection modulates pharmacokinetic variability. Although FcRn dysregulation was not directly measured in the AMP clinical trial, this correlation is consistent with cytokine-mediated suppression of *FCGRT*. Whether pre-existing immune dysregulation, rather than PrEP-associated mucosal injury, produces a similar pharmacokinetic effect is examined in the CAPRISA 012B sub-study below.

#### CAPRISA 012B: Baseline Immune Dysregulation Predicts bNAb Pharmacokinetic Variability

The CAPRISA 012B trial evaluated the safety and pharmacokinetics of two next-generation broadly neutralising antibodies VRC07-523LS and CAP256V2LS in HIV-negative women in South Africa provides a clinically significant association between inflammation and the FcRn axis [3]. While safety profiles were favourable, the trial identified transient lymphocytopenia in 19% of participants following bNAb administration [7]. This unique observation was absent in earlier North American cohorts. The affected women had significantly lower baseline IgG subclass concentrations and elevated macrophage inflammatory protein-1β (MIP-1β), TNF-α, IL-6, and IFN-γ compared to unaffected participants consistent with pre-existing immune dysregulation (Supplementary Table 1). Lower baseline IgG1 levels may reflect chronic FcRn saturation or accelerated catabolism modulated by sustained cytokine exposure, increasing susceptibility to accelerated bNAb clearance.

The findings suggest that participants who developed transient lymphocytopenia may have had a pre-existing immune dysregulation which contributes to interindividual variability in bNAb pharmacokinetics. Lower baseline IgG1 may reflect chronic FcRn saturation or accelerated IgG catabolism driven by sustained inflammatory cytokine exposure. These findings support the inflammation-FcRn hypothesis, as participants with elevated baseline inflammatory markers may exhibit reduced FcRn expression or function, leading to accelerated bNAb clearance and altered immune responses. Based on the CAPRISA 012B study findings, substantial interindividual variability in bNAb pharmacokinetics can exist within study cohorts, which correlates with baseline immunological and inflammatory states. Since neither AMP nor CAPRISA 012B clinical trials measured FcRn expression directly, this represents a missed opportunity for prospective clinical validation of the underlying hypothesis. Bridging these observations with cross-therapeutic evidence is therefore essential to justify integrating direct FcRn quantification into future bNAb trial designs.

#### Population Level Variability in bNAb Pharmacokinetics

Population pharmacokinetic analyses across several HIV prevention clinical trials have consistently identified body weight as a significant determinant of variability in bNAb pharmacokinetics [31, 32]. However, weight-adjusted analyses reveal persistent pharmacokinetic differences that cannot be attributed to distribution volume alone, suggesting additional biological determinants. In the AMP trials, sub-Saharan African women in HVTN 703/HPTN 081 exhibited shorter VRC01 half-lives compared to men who have sex with men and transgender individuals in HVTN 704/HPTN 085, even after adjustment for body weight [31]. Estimated VRC01 concentrations were lower in participants who subsequently acquired HIV than in those who remained uninfected.

The CAPRISA 012A clinical trial reported a substantially shorter VRC07-523LS half-life of 29 days following subcutaneous administration in South African women, compared to 42 day half-life observed in HVTN 127 [4]. The observed disparity surpasses pharmacokinetic predictions based on route of administration alone. These geographic and demographic variations suggest population-level differences in baseline immune activation and systemic inflammatory settings. Moreover, body weight may act as a surrogate for adiposity-associated inflammation, as adipose tissue constitutively produces TNF-α, IL-6, IL-1β, and monocyte chemoattractant protein-1 (MCP-1) in proportion to total adipose mass.

### Inflammation-mediated Pharmacokinetic Variability Across Therapeutic Fields

Cross-therapeutic pharmacokinetic evidence has consistently identified systemic inflammation as a key determinant of mAbs clearance. Table 1 summarises this evidence, the FcRn-dependent mechanisms operating in each clinical setting, and the lessons applicable to HIV-prevention bNAb clinical trials. Translating this evidence to bNAb pharmacokinetics must account for the lack of viral targets in seronegative cohorts. In the absence of circulating antigen load to facilitate complex-mediated clearance, target-mediated drug disposition (TMDD) remains minimal. FcRn-mediated IgG recycling, therefore, constitutes the dominant clearance pathway, making inflammation-driven FcRn dysregulation proportionally more consequential for bNAbs in prevention settings than in the therapeutic contexts from which the cross-therapeutic data are drawn.

Population pharmacokinetic analyses of FcRn-dependent mAbs across oncology, autoimmune, and infectious disease consistently identify CRP, serum albumin, and IL-6 as significant independent determinants of antibody clearance [33–35]. In pembrolizumab trials, lower serum albumin was identified as a significant independent determinant of mAbs clearance in population pharmacokinetic analyses from KEYNOTE clinical trials [36]. In rheumatoid arthritis and systemic lupus erythematosus, elevated endogenous IgG from polyclonal hypergammaglobulinaemia can saturate FcRn binding sites, potentially necessitating higher doses to achieve therapeutic concentrations [37]. These relationships were identified retrospectively, after dosing regimens had already been established, limiting their ability to inform dose selection. Underlying these associations at a receptor level, IFN-γ-mediated FCGRT transcriptional suppression reduces FCGRT mRNA by 40–60% under chronic immune activation, which can substantially reduce mAbs half-life [10]. The role of this mechanism in bNAb pharmacokinetics has not been directly quantified in HIV prevention cohorts. Prospective stratification by baseline inflammatory status, using high-sensitivity CRP, serum albumin, and total IgG, would enable the exposure-inflammation-efficacy analyses needed to justify phase 3 dosing decisions informed by individual biology rather than population-level assumptions.

## The Neonatal Fragment Crystallisable Receptor: Structure, Tissue Distribution and Regulation

### Structure and Assembly of the FcRn Complex

**Fig. 1:**
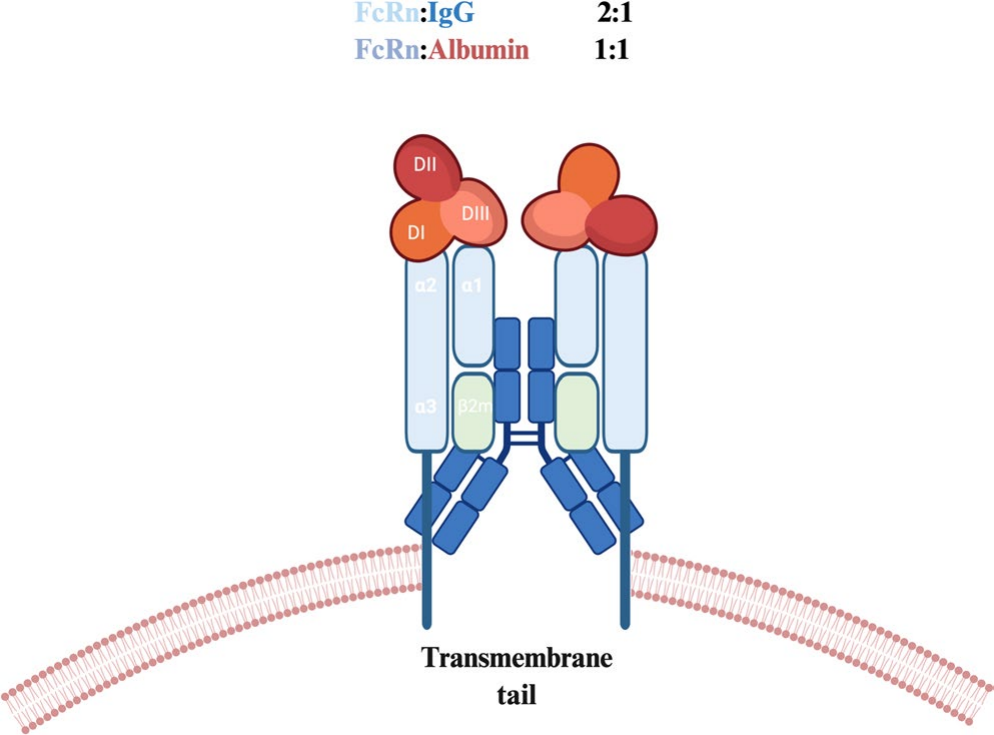
FcRn-IgG-albumin binding stoichiometry. Schematic representation of the neonatal Fc receptor (FcRn) showing simultaneous binding of IgG and albumin with distinct stoichiometries. FcRn (blue) binds two IgG molecules (red, 2:1 ratio) and one albumin molecule (orange, 1:1 ratio) through separate, non-competing binding sites. The receptor is anchored to the cell membrane via a transmembrane domain. This pH-dependent binding mechanism enables FcRn to rescue both IgG and albumin from lysosomal degradation, extending their serum half-lives. Created with BioRender.com

Understanding how inflammation modulates bNAb pharmacokinetics is essential for their effective application in HIV prevention. Central to this modulation is the FcRn, a heterodimeric glycoprotein that structurally resembles the major histocompatibility complex (MHC) class I molecule [38]. The functional receptor consists of a heavy α-chain encoded by the FCGRT gene on chromosome 19q13.33 and a 12-kDa beta-2-microglobulin (β2m) light chain encoded by the B2M gene on chromosome 15q21.1 [39, 40]. Despite structural homology to MHC class I, FcRn does not present peptide antigens. The groove between the α1 and α2 domains is occluded in FcRn, which instead promotes IgG Fc binding through contacts with the α2 and α3 domains and the β2m subunit (Fig. 1) [2, 41]. Stable FcRn expression requires non-covalent association with β2m [40]. In its absence, the FcRn heavy chain is retained within the endoplasmic reticulum (ER), where it forms “misfolded” disulfide-linked oligomers, which prevent its expression on cell surfaces, resulting in reduced ligand binding and transcytosis capacity [40].

The regulation of FcRn α-chain and β2m expression, therefore, becomes an important determinant of FcRn assembly, surface expression, and ligand recycling. This stoichiometric dependence of FcRn α-chain on β2m has significant implications under inflammatory conditions. β2m is a positive acute-phase reactant, and its upregulation during systemic inflammation may theoretically promote efficient FcRn heavy chain assembly. However, the impact of systemic inflammation on functional FcRn is determined by the homeostatic balance of β2m’s availability and cytokine-mediated regulation of the FCGRT heavy chain. This regulatory balance varies across different cell types and inflammatory stimuli, and it may not be fully reflected by the measurement of total FcRn protein abundance alone.

### FcRn Tissue Distribution

FcRn is expressed in a diverse range of tissue compartments and cell types, reflecting its dual roles in systemic IgG homeostasis and mucosal immunity. While our foundational understanding of FcRn function derives primarily from animal models, emerging evidence in humans suggests that FcRn may behave differently depending on the cell type. In vascular endothelial cells, FcRn is found predominantly within intracellular vesicular compartments, where it binds to IgG after receptor-mediated endocytosis and facilitates the pH-dependent recycling pathway [42–44]. In mucosal epithelial cells, which include the intestinal epithelium, respiratory airways, and the female genital tract (FGT), FcRn is similarly concentrated in intracellular endosomal compartments where it mediates bidirectional transcytosis of IgG across the epithelial barrier [45, 46]. This function is essential for establishing luminal IgG concentrations from parenterally administered mAbs. In haematopoietic cells, like monocytes, macrophages, dendritic cells, neutrophils, and B cells, FcRn is expressed at the cell surface as well as intracellularly, where it mediates the sorting and recycling of IgG immune complexes [43, 47]. The compartment-specific localisation of FcRn therefore establishes distinct pathways through which inflammatory modulation may differentially alter bNAb disposition across systemic, mucosal, and haematopoietic compartments. Consequently, serum concentration measurements alone may not fully reflect these compartment-specific effects.

### FcRn-mediated IgG Recycling and Homeostasis

**Fig. 2:**
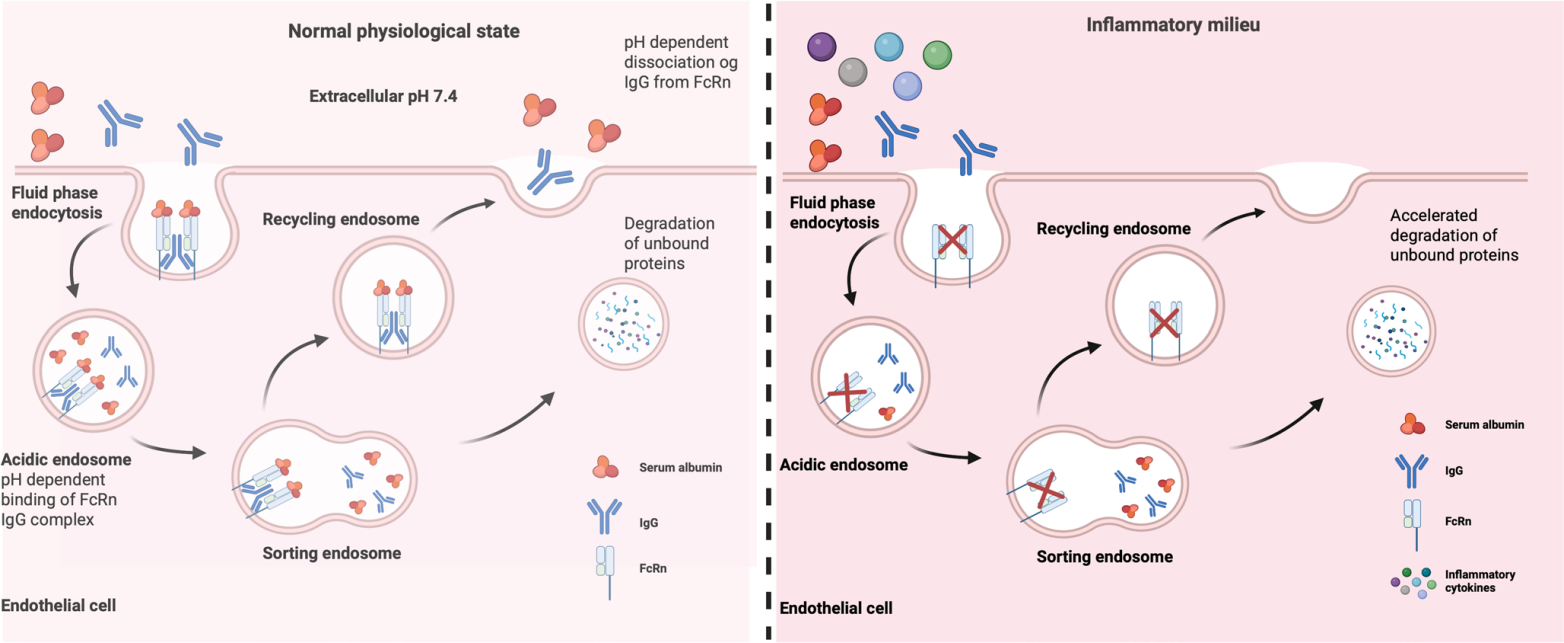
The FcRn-mediated recycling pathway for IgG and serum albumin under homeostatic (panel **A**) and inflammatory conditions (panel **B**). Panel **A**. Circulating IgG and serum albumin are internalised into endothelial cells through pinocytosis. Within the acidic endosomal compartment (pH 6.0-6.5), FcRn binds to both ligands, protecting them from lysosomal degradation. Unbound IgG and albumin are directed toward degradation pathways, while FcRn-bound IgG and albumin are sorted into recycling endosomes. These vesicles traffic back to the cell surface, where the neutral extracellular pH (7.4) promotes dissociation of IgG and albumin from FcRn, allowing them to be released back into systemic circulation. This process is key to the homeostatic maintenance and extended half-life of IgG and albumin in vivo. Panel **B.** Proinflammatory cytokines, including IFN-γ and TNF-α, suppress FCGRT transcription, thereby reducing FcRn availability. Diminished FcRn expression results in increased lysosomal degradation of internalised bNAbs, accelerating their clearance and reducing systemic exposure. Created with BioRender.com

Circulating IgG enters endothelial cells from serum through non-specific pinocytosis or receptor-mediated endocytosis. Within the acidic environment of endosomes (pH ~ 6.0), FcRn binds to the Fc region of IgG at the interface between the CH2 and CH3 domains (Fig. 2A) [39, 48]. This interaction involves key residues such as isoleucine 253 (I253) and the central histidine 310 and 435 (H310 and H435), which become protonated at low pH, facilitating high-affinity binding with FcRn residues glutamic acid 115 (E115) and aspartic acid 130 (D130), thus stabilising FcRn-IgG interaction [49, 50]. Once bound with the acidic endosome, the FcRn-IgG complex, which is based on a 2:1 stoichiometry (Fig. 1), where two FcRn molecules bind to the CH2-CH3 interfaces of a single IgG Fc dimer, is trafficked into recycling endosomes regulated by Rab4 and Rab11 GTPases [50, 51]. Upon re-exposure to the blood, an increase in pH to its physiological range of 7.4 leads to a gradual loss of protonation, resulting in the release of IgG from FcRn and demonstrating the pH-dependent nature of the FcRn-IgG interaction [49, 52]. Excess IgG molecules that do not form a complex with FcRn are trafficked to late endosomes and lysosomes through Rab7-mediated pathways for proteolytic degradation [42, 52].

FcRn’s saturation represents an underappreciated potential pharmacokinetic vulnerability for therapeutic bNAb. In individuals with hypergammaglobulinemia, commonly observed in chronic inflammatory, HIV infection, and endemic infectious disease settings, endogenous IgG may saturate FcRn binding sites, limiting receptor availability for therapeutic antibodies. In this context, the competitive advantage conferred by high-affinity Fc engineering may be limited by FcRn availability. While Fc-engineered variants like LS (M428L/N434S) and YTE (M252Y/S254T/T256E) increase FcRn affinity by 2–4 fold, their efficacy is still dependent on receptor abundance [53, 54]. Whether this advantage is fully preserved under chronic, LGSI remains to be defined. This competitive binding highlights a key bNAb pharmacokinetic vulnerability that is not fully accounted for in current pharmacokinetic models, warranting further systematic investigation.

FcRn also mediates the trafficking of IgG immune complexes (ICs), though their fate may depend on the size of the IgG-IC complex [55, 56]. Monomeric IgG-ICs can undergo FcRn-mediated recycling primarily in endothelial and epithelial cells and to a lesser extent in certain haematopoietic cell types [56, 57]. This allows for both IgG and its bound antigen to re-enter circulation. In contrast, multimeric IgG-ICs cannot be recycled by the FcRn and are directed to lysosomes for degradation [52, 57]. In conditions characterised by a high antigen load, such as chronic hepatitis B and C infections with high viremia, or autoimmune disorders like rheumatoid arthritis and systemic lupus erythematosus, with persistent autoantigen stimulation [58–61]. Under these conditions, therapeutic mAbs preferentially undergo lysosomal degradation as illustrated in Fig. 2B, which reduces their effective antibody concentrations and diminishes their therapeutic efficacy. The interplay between inflammation, FcRn, and ICs introduces further complexity in mAbs pharmacokinetics and clearance. The in vivo IC size thresholds that govern FcRn-mediated recycling or lysosomal degradation remain poorly defined. Similarly, the magnitude of FcRn saturation under clinically relevant inflammatory conditions and its impact on bNAb persistence remains poorly understood. Addressing these questions will be essential for developing pharmacokinetic models that reliably inform dosing strategies and patient stratification for bNAb-based therapies.

### Inflammatory Cytokine-mediated Regulation of FCGRT Transcription

**Fig. 3:**
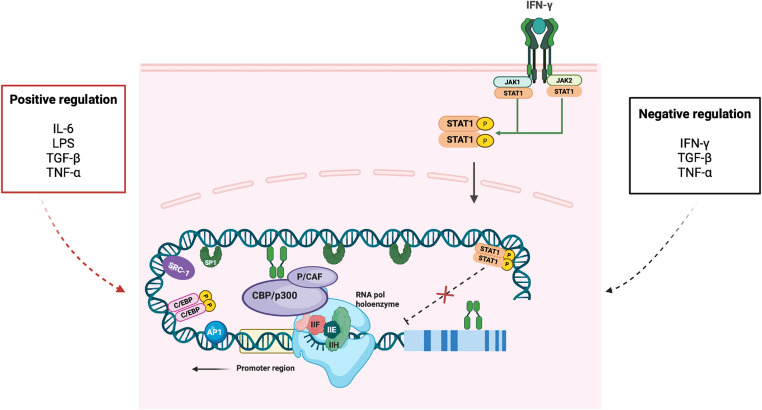
Transcriptional regulation of the FCGRT promoter under homeostatic and inflammatory conditions. Under homeostatic conditions, transcription factors including SP1, AP1, and phosphorylated C/EBP recruit coactivators CBP/p300 and P/CAF to the FCGRT promoter, facilitating RNA polymerase holoenzyme assembly and constitutive FCGRT transcription. IFN-γ signals through JAK1/JAK2 to phosphorylate STAT1, forming homodimers that bind the FCGRT promoter and sequester CBP/p300 from the transcriptional complex, blocking RNA polymerase assembly and suppressing FCGRT transcription. IL-6 and LPS upregulate FCGRT transcription through promoter-binding transcription factors. TNF-α exerts bidirectional effects on FCGRT expression, acting as a positive regulator in certain cell types and a negative regulator in others. Created with BioRender.com

FCGRT promoter activity is regulated by nuclear factor kappa B (NF-κB), activator protein 1 (AP-1), specificity protein 1 (SP1), and CCAAT/enhancer-binding protein (C/EBP) transcription factors, all of which are downstream targets of pro-inflammatory cytokine signalling (Fig. 3) [30, 62]. This transcriptional plasticity makes FcRn expression inherently responsive to an inflammatory milieu, unlike constitutively expressed housekeeping genes. In AGYW in SSA, several cytokines are chronically elevated and may consequently dysregulate FCGRT activity through distinctive transcriptional or post-transcriptional regulation. IFN-γ activates the canonical JAK1/2- Janus kinase-signal transducer and activator of transcription (JAK-STAT1) signalling pathway, resulting in STAT1 homodimerisation and nuclear translocation [9, 63]. STAT1 dimers bind to interferon-stimulated response elements (ISREs) in the FCGRT promoter and recruit transcriptional co-repressors, such as histone deacetylases (HDACs), resulting in chromatin tightening and transcriptional silencing [64].

**Fig. 4:**
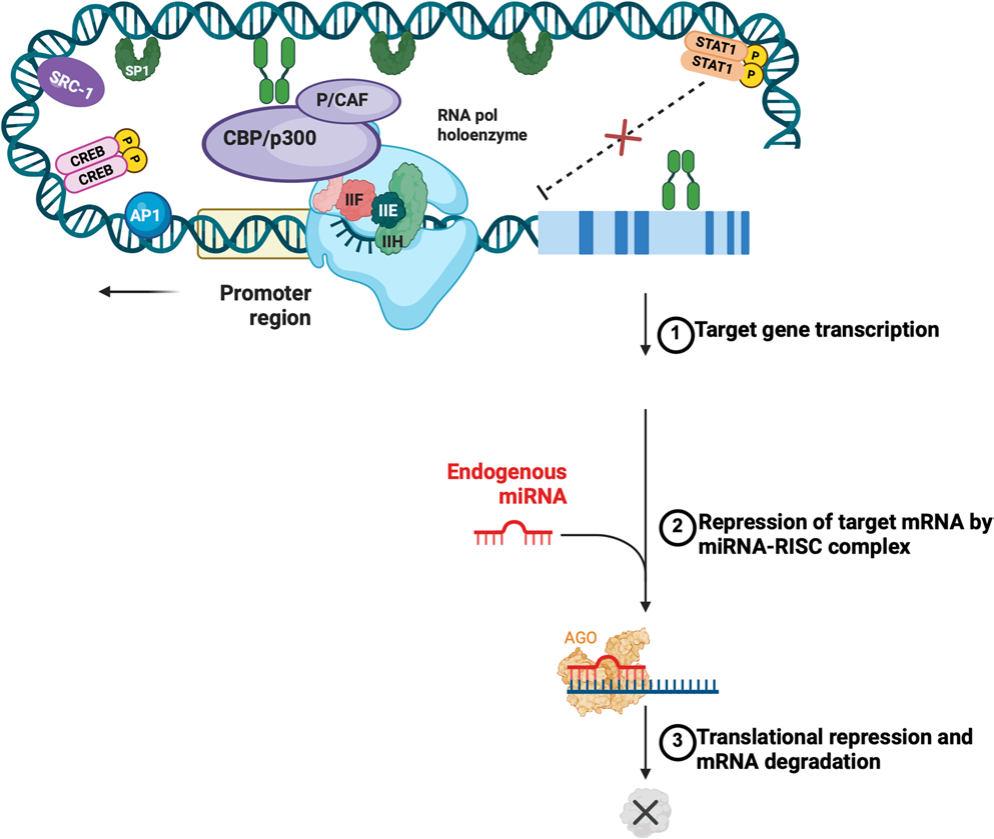
Post-transcriptional regulation of FCGRT mRNA by hsa-miR-3181. Following FCGRT transcription (step 1). hsa-miR-3181 binds complementary sequences in the FCGRT mRNA 3’-UTR (step 2), recruiting the Argonaute (AGO) protein complex to mediate mRNA degradation or translational repression (step 3), thereby reducing FCGRT mRNA expression. These mechanisms reduce FcRn availability, accelerating IgG catabolism and shortening therapeutic mAbs’ half-life. Created with BioRender.com

TNF-α exhibits context-dependent bidirectional effects on FCGRT transcription. In several cell types, TNF-α upregulates FCGRT transcription through NF-κB activation [10]. In human retinal epithelium cells, the same NF-κB pathway downregulates FcRn expression through recruitment of HDAC complexes to the p65 subunit [9]. This demonstrates that transcriptional direction is determined by the coregulatory protein environment rather than the signalling pathway alone. Moreover, microRNA hsa-miR-3181 has been demonstrated to reduce FCGRT mRNA expression by 43.5–51.3% in A549 lung epithelial cells, HEK293 embryonic kidney cells, and HepG2 liver carcinoma cells [39]. Whether miR-3181 expression is upregulated in an inflammatory milieu remains unestablished, representing an essential empirical gap (Fig. 4).

The regulatory plasticity of FCGRT observed across various tissues and cytokine profiles forms the basis of the bNAb pharmacokinetic hypothesis. This plasticity allows for a localised, responsive IgG recycling, which accounts for heterogeneity in antibody pharmacokinetics and systemic half-life. It also explains the interindividual variability observed in HIV prevention clinical trials, where individuals with higher inflammatory profiles exhibit faster IgG clearance. The translational gap does not reflect intrinsic biological variability but rather a lack of prospective measurement in HIV prevention cohorts.

### FCGRT Genetic Variations

Interindividual variability in FcRn expression is partially accounted for by a 37 bp variable number of tandem repeat (VNTR) polymorphism in the FCGRT promoter, which modulates transcriptional activity and influences IgG homeostasis [26]. The functional consequences of FCGRT polymorphisms on FcRn-mediated IgG trafficking remain poorly characterised. To date, five alleles (VNTR1-VNTR5) have been well established, each characterised by a different number of tandem repeats [26, 52]. Among these, VNTR2 and VNTR3 alleles are the most prevalent and clinically relevant [65]. Reporter gene assays have demonstrated that the VNTR3 allele is associated with significantly higher promoter activity compared to the VNTR2, resulting in elevated FCGRT mRNA levels and enhanced FcRn protein expression [65–67]. This enhanced FcRn expression leads to increased FcRn-IgG binding and recycling capacity, resulting in more efficient rescue of IgG from lysosomal degradation. Individuals carrying at least one copy of the lower-expressing VNTR2 allele exhibit reduced circulating IgG levels and increased IgG clearance rates, due to impaired FcRn-mediated recycling [65, 68]. This indicates an inherent pharmacokinetic vulnerability that exists independently of inflammatory status, but which may be exacerbated by systemic inflammation.

A significant yet underexplored gap in existing VNTR literature is the lack of data on allele frequencies in African populations, thus limiting our ability to assess population-specific implications on FcRn expression and IgG pharmacokinetics. Existing data on FCGRT VNTR polymorphisms have been generated primarily in Asian and European populations. Consequently, the prevalence of these variants in sub-Saharan African populations and the associated variation in FcRn expression remains poorly characterised. This limitation has important implications for the interpretation of bNAb pharmacokinetics beyond HIV prevention trials. A higher prevalence of the VNTR2 allele in African populations would imply lower baseline levels of FcRn expression in trial participants from high-burden settings. These variations could further modulate the inflammation-mediated regulation of FcRn described in this review. In clinical settings where mAbs are administered for autoimmune diseases and HIV prevention, the prevalence of the VNTR2 allele may lead to suboptimal IgG retention and diminished therapeutic efficacy due to impaired recycling functions. Targeted FCGRT genotyping studies in African cohorts participating in bNAb trials are therefore essential for direct translational relevance. Moreover, the FCGRT genotype could serve as a biomarker alongside inflammatory markers to guide bNAb dosing in future trial designs.

### FcRn-mediated Serum Albumin Regulation

FcRn rescues serum albumin from lysosomal degradation through the same intracellular salvage mechanism that governs IgG persistence. At endosomal pH 6.0-6.4, FcRn binds albumin domain III (DIII) through a 1:1 hydrophobic interaction, stabilised by protonation of H166 in the FcRn α1-domain [42, 69]. Deprotonation at physiological pH dissociates the complex and releases albumin into circulation [70]. Systemic inflammation disrupts albumin homeostasis through elevated vascular permeability, driving transcapillary leakage, and through FcRn suppression, impairing cellular recycling [71, 72]. The clinical consequence is hypoalbuminemia, which functions as both a biomarker of systemic inflammation and a direct indicator of compromised FcRn-mediated salvage. Serum albumin has been identified as a significant independent determinant of mAbs clearance across multiple therapeutic fields. In IBD, higher serum albumin concentrations are associated with lower infliximab clearance, consistent with shared FcRn-mediated salvage of albumin and IgG [73]. A physiologically based pharmacokinetic model of durvalumab further demonstrated that longitudinal albumin levels predict time-dependent changes in mAb clearance in the setting of cancer cachexia without requiring clinical PK data for fitting [74]. These findings establish a modelling framework directly applicable to the biomarker-guided dosing in SSA.

## Low-grade Systemic Inflammation in Sub-Saharan African Adolescent Girls and Young Women

Cytokine-mediated downregulation of FCGRT transcription and modulation of FcRn-mediated IgG recycling are relevant to SSA AGYW enrolled in HIV prevention clinical trials. These mechanisms reflect the chronic inflammatory milieu driven by endemic co-infections, STIs, and metabolic immune activation, manifesting as LGSI [11–13]. LGSI is characterised by a sustained increase in IL-1β, IL-6, TNF-α, and IFN-γ levels above homeostatic baselines [75]. This inflammatory profile is accompanied by increases in CRP, fibrinogen, and serum amyloid A, with a reciprocal decline in albumin without overt clinical illness [76, 77]. This persistent, asymptomatic state directly influences bNAb pharmacokinetics in HIV prevention settings. Despite its prevalence, LGSI remains unaccounted for in current bNAb dosing strategies for this population. The experimental evidence linking inflammation to FcRn suppression (Table 2) derives from models using acute, high-dose inflammatory stimuli. Whether sustained cytokine elevations characteristic of LGSI alter FcRn expression in vivo remains poorly defined. A quantitative dose-response relationship between inflammatory biomarker levels and bNAb clearance has not been established in any HIV prevention studies.

**Table 2:** Evidence of inflammation-modulated disruption of FcRn-mediated IgG homeostasis and bNAb pharmacokinetics

Mechanism	Molecular pathway	In vitro evidence	Animal model evidence	Clinical evidence	Evidence grade	Pharmacokinetic Consequence for bNAbs
**FCGRT transcriptional suppression through IFNγ/JAK-STAT1 pathway**	IFN-γ activates JAK-STAT1 pathway. Phosphorylated STAT1 binds the IFN-γ activation site in the FCGRT promoter, directly repressing transcription [9].	Dose-dependent suppression of FCGRT mRNA confirmed in IEC lines and THP-1 monocytes following IFN-γ stimulation [9].	FcRn-deficient mice demonstrate accelerated IgG clearance and reduced circulating IgG half-life, establishing that loss of FcRn protein function is sufficient to shorten IgG persistence [8, 78] No published murine model has directly induced FcRn suppression via IFN-γ stimulation.	Sustained IFN-γ elevation is well-documented in Helminth and STIs in high burden settings [79, 80]. Whether this translates to FCGRT suppression and reduced bNAb half-life in vivo has not been measured in any published clinical study.	**Grade II Moderate**	Pharmacokinetic variability dependent on magnitude of FCGRT suppression. Reduced endosomal recycling of internalised bNAb and accelerated lysosomal degradation of prophylactic and therapeutic mAbs.
**FCGRT modulation by TNF-α**	TNF-α activates NF-κB p65, which binds the FCGRT promoter. Transcriptional direction determined by cell-type-specific chromatin accessibility at NF-κB response elements and differential availability of transcriptional co-activators/co-repressors [10, 81].	NF-κB p65 activation downregulates FCGRT in retinal epithelium but upregulates it in THP-1 monocytes [10, 63]	Few models isolate TNF-α effect on FcRn independently of concurrent cytokines [10]	TNF-α is a hallmark of prevalent inflammatory conditions in SSA, acting as key modulator in both infectious and non-communicable diseases [75, 78]	**Grade III Limited**	Pharmacokinetic variability cannot be predicted without cell-compartment characterisation. If monocyte/macrophage upregulation dominates, FcRn recycling may be partially preserved. If epithelial downregulation dominates, lysosomal degradation accelerates.
**TGF-β1-mediated FCGRT upregulation**	JNK activation induces c-Jun binding to the AP-1 site in the FCGRT promoter of IEC [14].	IPEC-J2 cells [14]	Limited	In high-burden settings, high TGF-β1 levels have been documented in schistosomiasis, hypertension, and sickle cell disease [82, 83].	**Grade III Limited**	Upregulation does not translate to enhanced recycling under FcRn saturation.
**FcRn saturation caused by hypergammaglobulinaemia**	Elevated endogenous polyclonal IgG competes for FcRn, reducing receptor availability for therapeutic mAbs [84].	Competitive binding kinetics in cell-free SPR assays and FcRn-expressing epithelial cell transcytosis. IgG displacement under increasing polyclonal IgG quantified [85, 86].	In human Tg32 mice given high-dose IVIG (1 g/kg), wild-type mAb clearance increased markedly under competition, whereas Fc-engineered variants (YTE, LS) maintained their pharmacokinetic advantage [87]	Hypergammaglobulinaemia documented in SSA populations [88]; direct receptor displacement not measured in vivo	**Grade II Moderate**	Wild-type bNAb recycling impaired under FcRn saturation; effective half-life shortened. YTE/LS variants maintain clearance advantage through higher-affinity FcRn binding. Strongest PK rationale for prioritizing Fc-engineered bNAbs
**IgG immune complex**	Multimeric ICs exceed FcRn vesicular trafficking capacity; Rab7-mediated late endosome traffics to lysosome; monomeric ICs recycled via Rab4/Rab11 [89, 90]	IC size threshold characterised in endothelial and epithelial cell systems [60, 89].	Quantitative IC size thresholds under physiological inflammation not defined in vivo	Inference from chronic HBV/HCV viraemia and autoimmune IC formation contexts [56–59].	**Grade III Limited**	BNAbs bound to high-antigen-load multimeric ICs preferentially degraded; effective concentration reduced independently of dose or Fc engineering. Relevant under high viraemia or chronic antigen stimulation.
**Fc glycan remodelling (shift to G0 glycoforms)**	Inflammation suppresses B-cell galactosyltransferase and sialyltransferase activity; G0 accumulation destabilises CH2 domain, reduces FcγR and FcRn binding affinity [91, 92]	Galactosylation knockdown and enzymatic deglycosylation confirm reduced FcγR binding and altered FcRn engagement [93]	In inflammatory murine models, G0 IgG accumulation and accelerated catabolism have been confirmed [94–96]	In RA, G0 correlates with disease activity [58, 94, 96] Not measured in bNAb clinical trial participants	**Grade II Moderate**	Accelerated endogenous IgG catabolism occurs in inflammatory disease. bNAb glycan profiles are fixed at manufacture and not directly remodelled, though altered cellular recycling could plausibly affect clearance, but this remains unproven for bNAbs.

Each proposed mechanism was evaluated across three complementary lines of evidence and graded accordingly. Grade I represents strong evidence, supported by at least two independent in vitro studies, animal models, and consistent clinical correlation. Grade II indicates moderate evidence, with support from in vitro and animal studies and partial clinical association, although not yet demonstrated in HIV prevention contexts. Grade III reflects limited evidence, restricted to in vitro or animal data without clinical correlation. Grade IV denotes inferential evidence, derived from related biological systems without direct experimental validation of FcRn-inflammation interactions

Abbreviations: *AP-1* activator protein 1, *bNAbs* broadly neutralising antibodies; BeWo cells, human placental trophoblast cell line; c-Jun, component of activator protein-1 transcription factor, *FCGRT* neonatal Fc receptor gene, *FcRn* neonatal Fc receptor, *FcγR* Fc gamma receptor, *G0* glycoforms, agalactosylated immunoglobulin G glycoforms, *HBV* hepatitis B virus, *HCV* hepatitis C virus, *HEU* HIV-exposed uninfected, *IBD* inflammatory bowel disease, *IL-6* interleukin 6, *IEC* intestinal epithelial cell, *IFN-γ* interferon gamma, *IgG* immunoglobulin G, *IC* immune complex, *IPEC-J2 cells* porcine intestinal columnar epithelial cells, *IVIG* intravenous immunoglobulin, *JAK* Janus kinase; *JNK* c-Jun N-terminal kinase, *LGSI* low-grade systemic inflammation, *mAbs* monoclonal antibodies, *mRNA* messenger ribonucleic acid, *NF-κB* nuclear factor kappa beta, *PK* pharmacokinetics, *RA* rheumatoid arthritis, *STAT1* signal transducer and activator of transcription 1, *STIs* sexually transmitted infections, *SSA* sub-Saharan Africa, *TGF-β1* transforming growth factor beta 1, *THP-1 cells* human monocytic cell line, *TNF-α* tumour necrosis factor alpha, *Vd* volume of distribution, *VEGF* vascular endothelial growth factor

### Drivers of Chronic Immune Activation in Sub-Saharan African Population

The key drivers of chronic LGSI in SSA women are both intrinsic and extrinsic, reflecting an interplay between environmental exposures, genetic susceptibility, and pathogen-driven immune responses. Recurrent STIs caused by *Chlamydia trachomatis*, *Neisseria gonorrhoeae*, and *Trichomonas vaginalis* function as extrinsic triggers, modulating sustained persistent TNF-α and IL-1β elevation through TLR2 and TLR4 signalling in epithelial cells [97]. While these infections may present asymptomatically at a clinical level, they generate persistent subclinical inflammation characterised by activated lymphocyte infiltration and sustained IFN-γ production, resulting in transcriptional suppression of FCGRT in the female genital tract. This suppression impairs IgG transcytosis, thereby depleting mucosal IgG levels and bNAbs. Consequently, epithelial disruption and impaired IgG transport increase HIV acquisition risk 2.5-fold [79].

Beyond these localised bacterial triggers, the immunological landscape in endemic regions is shaped by chronic helminth infections (*Schistosoma mansoni* and soil-transmitted helminths) [80]. These parasites induce a mixed Th1/Th2 response characterised by elevated IL-6 and IL-10, which may dysregulate FcRn expression and alter IgG glycosylation patterns [98]. Concurrently, helminth-driven polyclonal B-cell activation produces hypergammaglobulinaemia, with elevated IgE, IgG1, and IgG4 levels [99]. This sustained elevation in circulating IgGs may saturate available FcRn binding sites, compounding the receptor dysregulation. In addition to these pathogen-driven mechanisms, intrinsic metabolic factors contribute significantly to LGSI.

Among AGYW in SSA who are clinically obese, adipose tissue drives constitutive secretion of TNF-α, IL-6, and IL-1β through macrophage infiltration and adipokine dysregulation [75]. This cytokine secretion induces systemic LGSI that differs from acute infection-driven inflammation but is equally capable of suppressing FcRn expression through the STAT1 and NF-κB pathways. Body weight has been consistently identified as a pharmacokinetic determinant in bNAb clinical trials. Still, weight-based dosing strategies account for bNAb distribution volume and not for adiposity-modulated FcRn suppression. Overweight AGYW may therefore remain pharmacokinetically underdosed even after weight-based dose adjustment. These drivers do not operate independently. Concurrent STI, helminth co-infection, and metabolic dysregulation activate partially distinct pathways to modulate FCGRT transcriptional suppression, FcRn saturation through hypergammaglobulinemia, and altered IgG glycosylation that converge on accelerated bNAb clearance as illustrated in Table 2.

## Antenatal bNAb Administration and Mother-to-Child Transmission Prevention

Beyond AGYW prevention, antenatal administration of bNAbs for the prevention of mother-to-child transmission (MTCT) of HIV represents a promising yet underexplored modality, particularly in high-burden settings like SSA. While maternal ART has significantly reduced HIV transmission rates, substantial gaps persist in the postnatal period through breastfeeding transmission and when maternal adherence to antiretrovirals (ARVs) is low. In the placenta, syncytiotrophoblast facilitates the selective transfer of maternal IgG during the third trimester when FcRn expression is upregulated, establishing neonatal passive immunity [38, 100]. This pathway is functionally selective. Although maternal plasma may be rich in albumin, IgG is far less abundant, yet syncytiotrophoblast FcRn preferentially transports IgG over albumin, ensuring that passive immunity is established in the neonate before maturation of their adaptive immune system [38, 101]. From an evolutionary perspective, syncytiotrophoblast FcRn may preferentially transport IgG as a protective mechanism. The indiscriminate transport of both FcRn ligands would potentially overwhelm FcRn’s trafficking capacity, deplete maternal oncotic pressure and predispose the mother to conditions like oedema and pre-eclampsia [51, 52]. The precise molecular mechanism for IgG’s preferential selectivity remains to be fully elucidated.

**Fig. 5:**
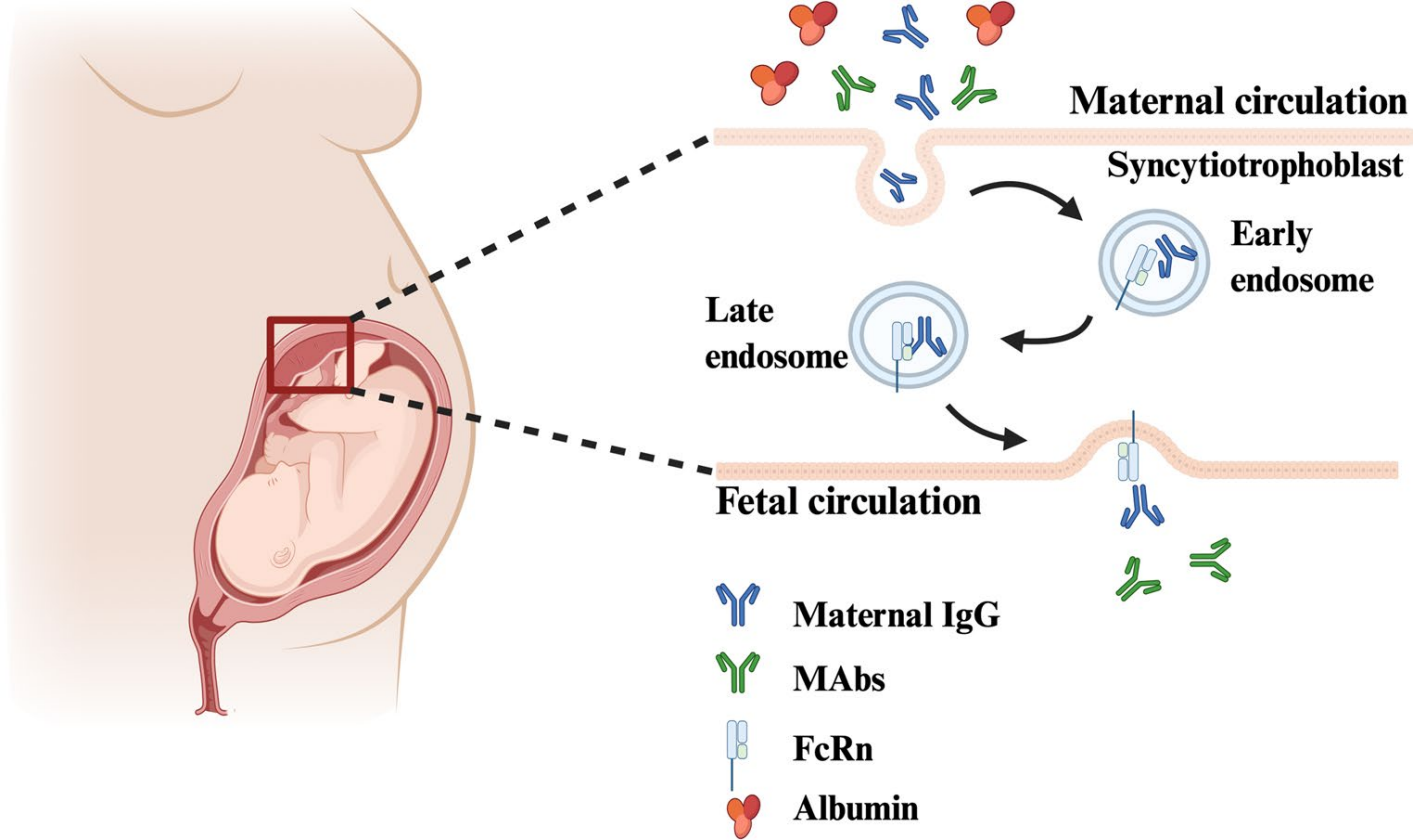
FcRn-mediated transport of maternal IgG and mAbs. Within the placenta, FcRn expressed on syncytiotrophoblast cells facilitates the selective transfer of maternal IgG and therapeutic mAbs from maternal circulation to foetal circulation. This receptor-mediated process provides passive immunity to the foetus and is also relevant to the transplacental pharmacokinetics of therapeutic mAbs. Created in BioRender https://BioRender.com/mcbytyg

Transplacental bNAb transfer is facilitated by this same FcRn-dependent mechanism, whose expression and function are vulnerable to the cytokine-driven suppression of FCGRT transcription illustrated in Fig. 5. Maternal conditions associated with systemic inflammation, including metabolic, autoimmune, and infectious diseases, elevate circulating TNF-α and IL-6, reduce syncytiotrophoblast FcRn expression and diminish transplacental IgG transcytosis. Consequently, bNAb dosing regimens designed to achieve protective maternal titres may be inadequate to guarantee adequate foetal bNAb exposure. In HIV-infected pregnant women with suboptimal viral suppression, the inflammation status simultaneously impairs maternal bNAb persistence and foetal bNAb transfer. This same inflammatory status has been shown to compromise neonatal innate immune responses, vaccine immunogenicity, and T-cell receptor repertoire development [102]. In HEU infants, inflammation-driven suppression of placental FcRn reduces foetal bNAb transfer when neonatal passive immunity is essential. Maternal bNAb concentrations meeting standard pharmacokinetic thresholds may not translate into adequate neonatal protection. The potential impact of this inflammation-FcRn mechanism warrants further investigation.

Physiologically based pharmacokinetic models that integrate placental FcRn maturation have been validated for IgG-class therapeutics during pregnancy [103]. These models have not incorporated inflammation as a covariate, nor have they been applied to bNAbs in HIV-infected pregnant women. The absence of inflammation-adjusted models represents an essential gap in the pharmacokinetic basis for antenatal bNAb dosing. Elucidating the relationship between maternal inflammatory burden, placental FcRn expression, and foetal bNAb concentrations is essential for evidence-based antenatal dosing. Whether Fc-engineered bNAbs incorporating YTE or LS modifications maintain their pharmacokinetic advantage under inflammation-mediated placental FcRn suppression remains unresolved. Characterising the relationship between maternal inflammatory burden, placental FcRn expression, and foetal bNAb concentrations is a prerequisite for evidence-based antenatal dosing in high-burden settings.

## Discussion and Future Perspectives

The transition of HIV bNAbs from experimental modalities to prophylactic or therapeutic successes depends on understanding the host-driven modulation of their effectiveness. Beyond molecular design, systemic inflammation poses a substantial barrier to bNAb efficacy. The evidence reviewed demonstrates that proinflammatory IFN-γ and IL-6 suppress FCGRT transcription, resulting in impaired FcRn-mediated IgG recycling. These findings, established in vitro and in murine models, are further supported by clinical observations in oncology and inflammatory bowel disease (Table 1). Clinical data from the AMP and CAPRISA 012 trials show that elevated inflammatory biomarkers correlate with accelerated bNAb clearance, a pattern consistent with FcRn dysregulation. However, neither trial directly measured FcRn expression nor stratified participants by inflammatory status. This association, therefore, remains inferential and hypothesis-generating rather than confirmatory. The premise that LGSI in SSA AGYW represents a persistent pharmacokinetic risk factor is biologically plausible and supported by the LGSI characterisation literature, but has not been tested in HIV prevention bNAb pharmacokinetic studies. The VNTR-inflammation interaction hypothesis is biologically consistent and supported indirectly by IBD data, but remains untested in SSA cohorts or under experimental conditions combining genotype with cytokine exposure.

The populations who stand to benefit most from long-acting bNAb prevention, mainly AGYW in SSA, pregnant women at risk of vertical transmission, and HEU infants, are precisely those in whom co-endemic infections, STIs, and metabolic immune activation converge to create an unfavourable pharmacokinetic environment. BNAbs with enhanced FcRn binding affinity, neutralisation and potency are necessary but inadequate if the FcRn expression is suppressed by an activated immune state in the individuals they are designed to protect. Current HIV prevention trials have evaluated safety, pharmacokinetics, and protective efficacy, with dosing strategies anchored to body weight and therapeutic drug monitoring. Inflammatory status has not been incorporated as a pharmacokinetic determinant, nor have participants been stratified by inflammation status at enrolment. Variability in inflammatory status may therefore contribute to interindividual differences in bNAb exposure unaccounted for by weight-based dosing, potentially explaining a portion of the pharmacokinetic heterogeneity observed across recent trials.

This review, therefore, underscores a knowledge gap rather than a null finding. The inflammation-pharmacokinetic axis has not been disproven; instead, it has not been evaluated under conditions designed to detect it. Current HIV prevention clinical trials have primarily focused on evaluating safety, pharmacokinetics, and protective efficacy, with dosing strategies largely based on body weight and TDM. These clinical trials and studies have not incorporated inflammatory status as a pharmacokinetic determinant or stratified participants by inflammatory status at enrolment. Variability in inflammatory status may therefore contribute to differences in bNAb exposure that are not accounted for by weight-based dosing or TDM, potentially explaining some of the pharmacokinetic heterogeneity observed in recent HIV prevention trials. Incorporating baseline inflammatory profiling and mucosal biomarker sampling into future trials may improve the interpretation of exposure-response relationships and help ensure that pharmacokinetic data are representative of populations at highest risk of HIV acquisition.

### Biomarker-guided and Personalised Dosing Frameworks

The era of one-size-fits-all for HIV bNAb dosing may be ending. If systemic inflammatory status is a key determinant of bNAb clearance, then baseline inflammatory biomarkers should inform dosing decisions. This is applicable at both the population level, in determining standard doses and dosing intervals for high-burden settings, and at the individual level, to identify participants who require dose adjustment to maintain protective concentrations. However, this approach requires substantial validation before clinical implementation. High-sensitivity CRP represents a clinically relevant biomarker given its associations with mAbs clearance in other therapeutic areas. Whether specific CRP thresholds can predict bNAb clearance in HIV prevention populations remains to be established through prospective studies. Similarly, serum albumin consistently correlates with immunoglobulin half-life across therapeutic contexts, potentially reflecting FcRn-mediated recycling capacity. However, validation in bNAb populations is needed. Total serum IgG concentrations may also introduce binding competition on FcRn recycling, particularly in populations with chronic immune activation, though the clinical significance of this mechanism for bNAb dosing requires investigation. Additional biomarkers such as IL-6, I-FABP, and FCGRT genotyping may improve identification of individuals at risk of accelerated bNAb clearance, but their predictive value remains largely theoretical.

Developing biomarker-based dosing strategies that are both precise and feasible represents a significant challenge in HIV prevention programmes. Future research should prioritise identifying a minimal set of validated biomarkers that capture clinically meaningful variability in bNAb clearance while remaining feasible for implementation in resource-limited settings. Moreover, longitudinal pharmacokinetic modelling studies that incorporate these biomarkers alongside FcRn VNTR genotype, accounting for the under-characterised distribution of VNTR2 and VNTR3 alleles in African populations, would provide the empirical basis for a stratified dosing algorithm that could be implemented within existing HIV prevention programme infrastructure. Until such validation is achieved, biomarker-guided dosing remains a promising but unproven strategy for optimising bNAb pharmacokinetics and efficacy across diverse populations.

## Conclusion

Systemic inflammation represents an essential yet underexplored determinant of bNAb pharmacokinetics in HIV prevention. Emerging clinical trial data demonstrate that inflammatory biomarkers correlate with accelerated clearance and reduced efficacy. Cross-therapeutic evidence establishes that inflammatory cytokines suppress FcRn expression, saturate receptor binding capacity, and redirect antibodies to degradation pathways. In sub-Saharan African populations, the convergence of STIs, helminth co-infections, and metabolic immune activation creates chronic immune activation that current dosing strategies do not account for. To address this gap, prospective inflammatory biomarker profiling must be incorporated into phase 2/3 trials to validate pharmacokinetic relationships and identify high-risk subgroups. These findings can then inform the engineering of inflammation-resilient Fc variants designed to maintain their pharmacokinetic advantage under FcRn suppression. These insights will enable the implementation of biomarker-guided dosing algorithms that adjust treatment intervals based on an individual’s specific inflammatory burden, therefore enhancing bNAb pharmacokinetics and efficacy across diverse populations.

## Supplementary Information

Below is the link to the electronic supplementary material.

Supplementary Material 1

## Data Availability

No datasets were generated or analysed during the current study.

## Glossary

1. C-reactive protein (CRP): A protein produced by the liver in response to inflammation. It is often used as a biomarker to assess inflammation or infection in the body.
2. Broadly neutralising antibodies (bNAbs): Specialised antibodies capable of neutralising a wide range of HIV strains. They are a focus of research for vaccines and therapies to prevent or treat HIV.
3. Human Immunodeficiency Virus (HIV): A virus that attacks the immune system, specifically CD4 cells (T cells), making the body vulnerable to infections.
4. Immunoglobulin G (IgG): The most abundant antibody in the blood and extracellular fluid. It plays a crucial role in immune defence against infections and is often used in therapeutic antibody research.
5. Monoclonal Antibodies (MAbs): Laboratory-made proteins designed to mimic the immune system’s ability to fight off harmful pathogens, such as viruses or bacteria. They are commonly used in research and treatment, including HIV.
6. Neonatal fragment crystallisable receptor (FcRn): A receptor involved in prolonging the half-life of IgG antibodies and albumin in the bloodstream. It is also important for the transfer of maternal antibodies to the foetus during pregnancy.
