# Supplementary material for: Systemic Inflammation as a Modulator of FcRn-dependent IgG Pharmacokinetics: Implications for Broadly Neutralising Antibody Efficacy in HIV Prevention
Source: Curr HIV/AIDS Rep. 2026 Jul 10;23(1):23. doi: 10.1007/s11904-026-00791-2 (PMC13354693; doi:10.1007/s11904-026-00791-2)
Supplement: Supplementary file 1 — Supplementary Material 1 [file 11904_2026_791_MOESM1_ESM.docx]

Supplementary table 1: Clinical landscape of HIV prevention bNAbs and key pharmacokinetic findings

| Clinical trial ID | bNAb(s) | Epitope target | Population | Location | Observed half-life (days) | Trial status | Key pharmacokinetic findings | Reference |
| --- | --- | --- | --- | --- | --- | --- | --- | --- |
| NCT02165267 | VRC01 | CD4 binding site | HIV-uninfected adults (n=84) | United States of America (USA) | ~15 days (IV)  ~17 days (SC; 74% SC bioavailability) | **Completed** | Population PK model (n=1,117) established CL=0.40 L/day and Vd=3.6 L, with body weight as a significant positive covariate for both parameters; SC bioavailability was 74% with Ka=0.24/day and Tmax of 2-3 days. | Huang et al, 2017 |
| NCT02568215 | VRC01 | CD4 binding site | Cisgender women (n=1,924) | Botswana, Kenya, Malawi, Mozambique, South Africa, Tanzania and Zimbabwe | ~13 days (IV). Shorter than HVTN 704 after weight adjustment | **Completed** | Primary efficacy 0% overall (HR 0.75, 95% CI 0.46-1.22; p=0.32) but 75% against VRC01-sensitive strains (IC₈₀ <1 µg/mL). A PK pilot sub-study demonstrated shorter mean peripheral Vd and elimination t½ in SSA women versus HVTN 704 MSM after weight adjustment, with residual clearance differences unexplained by weight, age, or CD4 count. | Huang et al, 2021 |
| NCT02716675 | VRC01 | CD4 binding site | MSM & transgender women (n=2,699) | Brazil, Peru, Switzerland and USA | ~17 days (IV). Higher than HVTN 703 after weight adjustment | **Completed** | I-FABP post-hoc sub-study in PrEP users demonstrated 0.08 L/day faster VRC01 clearance, 0.29 day/mL lower dose-normalised AUC, and an estimated 14% reduction in efficacy against VRC01-sensitive strains, independent of CD4 count and viral load. | Huang et al, 2021 |
| NCT02511990 | 10-1074 | V3 glycan | HIV-uninfected (n=14) & PLWH (n=19) | USA | ~24 days in HIV uninfected  ~13 days PLWHIV | **Completed** | Single IV infusion (3-30 mg/kg) produced t½=12.8 days and maximal viral load decline of 1.52 log₁₀ copies/mL; rapid selection of pre-existing V3 loop resistance mutations (N332 glycan loss) occurred in all viraemic recipients under monotherapy. | Caskey et al, 2017 |
| NCT02960581 | PGT121 | V3 glycan | HIV-uninfected (n=20) PLWH on ART (n=15) Viraemic PLWH (n=13) | USA | ~22 days in HIV-uninfected  ~16 days in PLWHIV on ART  ~14 days in PLWHIV with viraemic | **Completed** | In HIV-uninfected adults, median t½=22 days (range 16-29) with dose-specific estimates of 18.8, 22.9, 23.0 (IV 3, 10, 30 mg/kg), and 20.9 days (SC 3 mg/kg). AUC was greater in HIV-uninfected than HIV-infected participants at equivalent doses, and shorter t½ in viraemic participants was attributed to sink-effect removal of PGT121-virus immune complexes. | Stephenson et al, 2021 |
| NCT02599896 | VRC01LS | CD4 binding site | HIV-negative adults (n=25) | USA | 71 ± 18 days (IV) 4.7-fold extension vs VRC01 (~15 days) | **Completed** | t½=71 ± 18 days with CL=36 mL/day the longest observed for any CD4 binding site class bNAb . | Gaudinski et al, 2018 |
| NCT03015181 | VRC07-523LS | CD4 binding site | HIV-negative adults (n=26) | USA | 38 days (IV; 1-40 mg/kg) 33 days (SC; 5 mg/kg) | **Completed** | t½=38 days (IV) and 33 days (SC) which was ~5-fold greater in vitro potency than VRC01 with 95% global HIV-1 breadth. SC 5 mg/kg concentrations exceeded the estimated PT80 threshold through week 24, directly informing the 20 mg/kg SC dose selection in CAPRISA 012A. | Gaudinski et al, 2019 |
| CAPRISA 012A | VRC07-523LS + PGT121 | CD4 binding site + V3 glycan | HIV-negative women (n=45) | KwaZulu-Natal, SA | VRC07-523LS: 29 days (SC 20 mg/kg) ~31% shorter than HVTN 127 (42 days)  PGT121: ~20 days (SC 10 mg/kg) ~9% shorter than US comparator | **Completed** | VRC07-523LS SC 20 mg/kg demonstrated a median concentrations of 9.65 µg/mL at week 16 and 3.86 µg/mL at week 24. PGT121 SC 20 mg/kg was 1.37 µg/mL at week 16; SC well tolerated with 86.7% injection-site acceptability for 2-3 doses/year, no anti-drug antibodies. | Mahomed et al, 2022 |
| CAPRISA 012B | CAP256V2LS ± VRC07-523LS ± PGT121 (SC & IV; mono & combos) | V1V2 apex + CD4 binding site+ V3 glycan | HIV-negative and HIV+ women (n=42) | KwaZulu-Natal, SA | CAP256V2LS: 43 days SC  VRC07-523LS: 66 days SC | **Completed** | No serious adverse events or dose-limiting toxicities; 19% (8/42) developed transient lymphocytopenia on day 1 accompanied by neutrophilia and decreased eosinophils/monocytes; commonly reported reactogenicity: headache (91% SC), chills (74% SC), malaise/fatigue (56% SC); subcutaneous administration at 10-20 mg/kg with EDP achieved protective concentrations (CAP256V2LS ≥1 μg/mL, VRC07-523LS ≥10 μg/mL) maintained through 24 weeks; co-administration increased CAP256V2LS concentrations; both antibodies retained functional neutralization activity post-infusion. | Mahomed et al, 2023 |
| NCT03928821 | PGT121 + PGDM1400 + 10-1074 + VRC07-523LS (dual & triple IV) | V3 glycan + V1V2 apex + CD4bs | HIV-negative adults (n=27) | USA | VRC07-523LS: ~42 days PGDM1400: ~24 days  PGT121: ~22 days  10-1074: ~13 days | **Completed** | No significant PK differences between dual vs triple combinations. Safe and well tolerated; no serious adverse events related to study products; no pharmacokinetic interactions among bNAbs in dual or triple combinations. Triple combination (PGDM1400+PGT121+VRC07-523LS) achieved greater neutralisation magnitude and breadth than dual combinations (AUC day 28: T4=3.37 vs T1-T3=2.9-3.0). | Sobieszczyk et al, 2023 |
| NCT04212091 | PGT121.414.LS ± VRC07-523LS (IV & SC; first-in-human) | V3 glycan (LS) + CD4bs | HIV-negative adults (n=33) | USA | PGT121.414.LS: 71 days, 3-fold extension vs parental PGT121 (~22 days)  VRC07-523LS: 53 days | **Completed** | First-in-human PGT121.414.LS across IV 3–30 mg/kg and SC 5 mg/kg. The LS mutation delivered a ~3-fold t½ extension versus parental PGT121 (71 vs ~22 days); SC bioavailability 86.1%. No serious adverse events; SC combination regimens with VRC07-523LS were well tolerated. | Edupuganti et al, 2025 |

**Abbreviations:** ADA, anti-drug antibodies; AGYW, adolescent girls and young women; AMP, Antibody Mediated Prevention; ART, antiretroviral therapy; ATI, analytical treatment interruption; AUC, area under the concentration-time curve; bNAb, broadly neutralising antibody; CD4bs, CD4 binding site; CL, clearance; CRP, C-reactive protein; FcRn, neonatal Fc receptor; HPTN, HIV Prevention Trials Network; HVTN, HIV Vaccine Trials Network; I-FABP, intestinal fatty acid binding protein; IFN-γ, interferon-gamma; IgG, immunoglobulin G; IL, interleukin; IM, intramuscular; IV, intravenous; Ka, first-order SC absorption rate constant; LGSI, low-grade systemic inflammation; LS, Met428Leu/Asn434Ser Fc half-life extension mutations; MPER, membrane-proximal external region; MSM, men who have sex with men; PK, pharmacokinetics; popPK, population pharmacokinetics; PT80, 80% inhibitory serum titre at which 80% of susceptible viruses are neutralised; SA, South Africa; SC, subcutaneous; SSA, sub-Saharan Africa; t½, elimination half-life; TG, transgender; TNF-α, tumour necrosis factor alpha; V1V2, variable loops 1 and 2; V3, variable loop 3; Vd, volume of distribution; YTE, Met252Tyr/Ser254Thr/Thr256Glu Fc half-life extension mutations.
